# Massive blood transfusion for obstetric haemorrhage

**DOI:** 10.1186/cc12308

**Published:** 2013-03-19

**Authors:** S Simmons, WE Pollock, L Phillips, S McDonald

**Affiliations:** 1 Mercy Hospital for Women, Heidelburg, Australia; 2 Monash University, Prahran, Australia; 3 La Trobe University, Bundoora, Australia

## Introduction

Obstetric haemorrhage remains a leading cause of maternal mortality and severe morbidity. Cardiovascular and haemostatic physiology alters in pregnancy and massive transfusion protocols have been implemented for obstetric haemorrhage based on limited evidence. The objective of this study was to examine the pattern and rate of blood products used in massive transfusion for obstetric haemorrhage in a tertiary obstetric hospital.

## Methods

Massive transfusion was defined as 5 or more units of red blood cells within 4 hours in accordance with the Australian Massive Transfusion Registry definition. Following ethics approval, all cases filling this criterion were identified in the hospital's birthing and blood bank systems. Data were extracted from the medical histories and analysed using SPSS. *P <*0.05 was considered statistically significant.

## Results

Twenty-eight women in three years (2009 to 2011) underwent a massive transfusion for obstetric haemorrhage, with nine receiving more than 10 units of RBCs in 24 hours. Eleven (39%) were admitted to the ICU and 11 underwent a hysterectomy, of which six were admitted to the ICU. The median estimated blood loss was 4,335 ml (IQR 3,000 to 5,000). Median blood product delivery was RBC 8 units (IQR 6 to 13); FFP 4 units (IQR 4 to 8); platelets 4 units (IQR 0 to 8) and cryoprecipitate 3 units (IQR 0 to 10). One-half of the women received the first four units of RBCs in less than 34 minutes. Other blood products were started a median of 49 minutes, 44 minutes and 75 minutes after the RBC transfusion commenced, respectively. Eight women had a fibrinogen level <0.8 g/l on the initial coagulation test during the haemorrhage. The remaining 20 women had a median fibrinogen level of 3.7 g/l (IQR 3.15 to 4.9). There was no difference in the transfusion of RBCs (*P *= 0.20), FFPs (*P *= 0.96) and platelets (*P *= 0.48) in women who showed an initial low fibrinogen and those who did not, although there was a difference in the number of units of cryoprecipitate (*P <*0.05). The median lowest Hb during the haemorrhage was 66 g/l (IQR 51 to 80) and median discharge Hb was 103 g/l (IQR 96 to 113). No blood product reaction was noted and there was one death.

## Conclusion

Massive transfusion for obstetric haemorrhage involved rapid blood product administration with no consistent pattern in the ratio of products administered.

